# The Ubiquitous Wilt-Inducing Pathogen *Fusarium oxysporum*—A Review of Genes Studied with Mutant Analysis

**DOI:** 10.3390/pathogens13100823

**Published:** 2024-09-24

**Authors:** Edan Jackson, Josh Li, Thilini Weerasinghe, Xin Li

**Affiliations:** 1 Michael Smith Laboratories, University of British Columbia, Vancouver, BC V6T 1Z4, Canada; 2 Department of Botany, University of British Columbia, Vancouver, BC V6T 1Z4, Canada

**Keywords:** plant fungal pathogen, Fusarium wilt, *Fusarium oxysporum*, fungal pathogenesis, fungal growth, conidiation, mutant analysis, knockout

## Abstract

*Fusarium oxysporum* is one of the most economically important plant fungal pathogens, causing devastating Fusarium wilt diseases on a diverse range of hosts, including many key crop plants. Consequently, *F. oxysporum* has been the subject of extensive research to help develop and improve crop protection strategies. The sequencing of the *F. oxysporum* genome 14 years ago has greatly accelerated the discovery and characterization of key genes contributing to *F. oxysporum* biology and virulence. In this review, we summarize important findings on the molecular mechanisms of *F. oxysporum* growth, reproduction, and virulence. In particular, we focus on genes studied through mutant analysis, covering genes involved in diverse processes such as metabolism, stress tolerance, sporulation, and pathogenicity, as well as the signaling pathways that regulate them. In doing so, we hope to present a comprehensive review of the molecular understanding of *F. oxysporum* that will aid the future study of this and related species.

## 1. Introduction

The *Fusarium* genus contains a diverse range of filamentous ascomycete fungi widely distributed in the soil and associated with plants. While *Fusarium* strains are predominantly non-pathogenic and often form mutualistic relationships with host plants, the genus also includes some of the most important pathogens of both plants and animals. The genus is highly diverse, with approximately 300 phylogenetically distinct species, many of which have been grouped into monophyletic species complexes [1]. Amongst them, *Fusarium oxysporum* Schltdl. emend. Snyder & Hansen is considered the most economically important, containing the most plant pathogenic strains in the genus [2].

Plant pathogenic strains of *F. oxysporum* cause Fusarium wilt diseases in many crop species, including several economically important crops such as tomato, banana, sweet potato, onion, and legumes [3]. In addition, they can infect ornamental plants such as tulips, carnations, and orchids. Fusarium wilt displays characteristic symptoms, including browning of the vascular tissues and drooping of the older leaves, followed by necrosis, defoliation, and death of the entire plant.

The taxonomy of *F. oxysporum* has proved difficult to resolve. A very broad definition of the species has been adopted, encompassing significant genetic and morphological diversity [4]. Reflecting this, *F. oxysporum* is generally referred to as a species complex, and future taxonomic revisions within this complex are needed to resolve the complexity as more genetic evidence becomes available. Although the *F. oxysporum* species complex (FOSC) has a broad host range, individual strains tend to specialize on a small number of hosts, even to the level of a specific cultivar. Consequently, pathogenic strains of *F. oxysporum* are typically categorized based on host specificity by the informal taxonomic ranking *formae specialis* (f. sp.), with over 120 *formae speciales* described [1]. Genealogical studies have revealed that many *formae speciales* of *F. oxysporum* are not monophyletic, indicating that pathogenicity to certain host species has evolved independently multiple times [5,6,7]. This conclusion has important implications for the study of *F. oxysporum* biology and the development of disease control strategies, as different clonal lineages may depend upon different pathogenicity factors to infect the same host. Despite this, the *formae specialis* classification is widely used by plant pathologists as a useful and efficient system for describing and studying the pathogenicity of *F. oxysporum* isolates, although caution must be exercised when generalizing results to non-monophyletic groupings.

Even within the same *formae speciales*, different *F. oxysporum* strains can display differing virulence on different cultivars of the same host species. In these cases, the *formae specialis* is further subdivided into pathogenic races, with each race specializing on a different host cultivar [6]. *F. oxysporum* isolates are also categorized into vegetative compatibility groups (VCGs), defined as groupings of isolates that can fuse to form stable heterokaryons [7]. This distinct classification appears to better reflect phylogenetic relatedness than the *formae speciales* system [8].

*F. oxysporum* virulence is mediated through its diverse repertoire of pathogenicity factors that facilitate invasion and colonization, including cell wall-degrading enzymes (CWDEs), mycotoxins, and secreted effectors [9,10,11]. Many of these pathogenicity factors form host-specific gene-for-gene interactions, in which pathogen effectors interact with host immune receptors locked in an evolutionary arms race. These interactions often play important roles in determining the high degree of host specificity exhibited by *F. oxysporum* strains [12,13].

Like many *Fusarium* species, *F. oxysporum* is anamorphic, with no observed sexual stage [14]. It produces three types of asexual spores, macroconidia, microconidia, and chlamydospores, in a process known as conidiation [15]. Macroconidia are long, sickle-shaped, multinucleate with multiple septa, and are produced from conidiophores in specialized structures known as sporodochia. Microconidia are smaller, oval-shaped uninucleate spores produced by conidiophores on the mycelium. Chlamydospores are small, round, thick-walled spores produced by vegetative hyphae or from macroconidia [2]. While conidia and even mycelia may be able to survive in the soil for short periods, chlamydospores are the most resistant form; they are believed to be the main vessel of *F. oxysporum* persistence in the soil [16].

**Figure 1:**
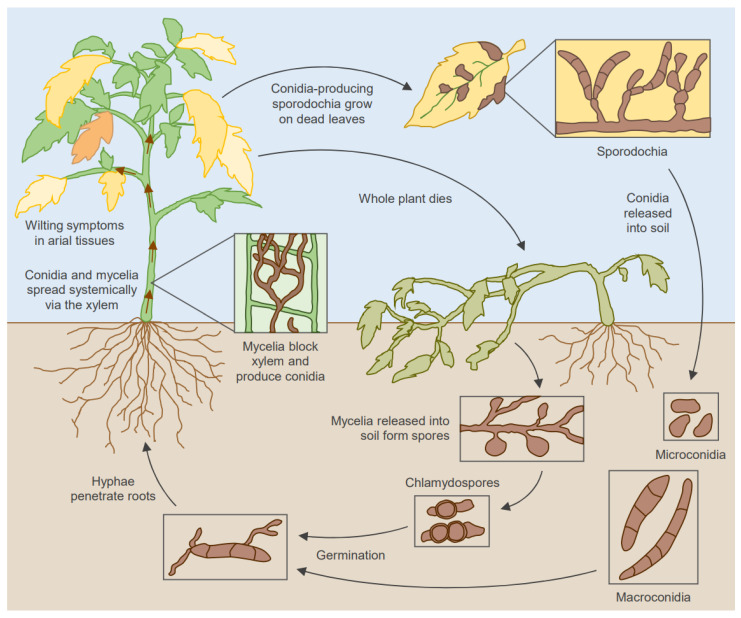
Lifecycle and disease cycle of *F. oxysporum*.

Spore germination is triggered upon favorable temperature and humidity, and they can grow into hyphae to penetrate the host root epidermis [9] (Figure 1). Once initial infection has been established, hyphae can grow intercellularly through the root cortex to the vascular tissues and enter the xylem. In the xylem, hyphae produce macro- and microconidia, which are then transported to the above-ground tissues to cause systemic infection [17]. Hyphae can grow to form a thick mycelium, blocking the xylem and leading to browning of the vasculature and wilting and chlorosis of the leaves and stems. The fungus can rapidly proliferate on dying host tissue, producing spores that can survive in soil to infect new plants. While primarily considered a soilborne pathogen, certain *F. oxysporum* strains produce airborne conidia from the surfaces of infected tissues, aiding systemic colonization and dispersal to new soils [18,19,20]. Spores have even been found to be dispersed via insect vectors such as shore flies, allowing dispersal over long distances [21].

Due to its ability to persist in the soil for long periods, *F. oxysporum* is difficult to control. Whilst fungicides such as carbendazim, chloropicrin, and 1,3-dichloropropene have been effective, there are many concerns with their use, including harm to beneficial microbes, disruption of aquatic ecosystems, and the development of fungicide resistance [22]. Management practices such as crop rotation and destruction of infected plants have been used with some success in reducing soil inoculum, but the long persistence of chlamydospores makes such strategies difficult to sustain [23]. Future management will also find it challenging to deal with the development of fungicide resistant cultivars.

In this review, we provide an update on recent developments in the molecular biology of *F. oxysporum*, with a focus on mutant analysis. Mutant analysis is a powerful tool for the study of fungal biology and establishing a causal relationship between genes and their biological functions. Several techniques have been utilized for the generation of *F. oxysporum* mutants, including homologous recombination (HR)-based gene deletion, RNA interference (RNAi), and T-DNA insertion. We begin with an overview of the *F. oxysporum* genome and the insights provided by genomic, transcriptomic, and secretomic analyses. We then discuss key molecular findings from the *F. oxysporum* genes that have been studied through mutant analysis to provide a comprehensive synthesis of the molecular genetic work that has taken place in recent decades.

## 2. The Features of the *F. oxysporum* Genome

### 2.1. Genome Sequences

The reference genome of *F. oxysporum* f. sp. *lycopersici* (*Fol*) 4287 (race 2, VCG 0030), isolated from tomato (Murcia, Spain), was first generated with Sanger sequencing with 6.8× coverage [24]. Using 114 scaffolds with an N_50_ scaffold length of 1.98 Mb, the *Fol* strain was determined to have 15 chromosomes comprising approximately 59.9 Mb (GenBank accession number: AAXH01000000, https://www.ncbi.nlm.nih.gov/nuccore/AAXH00000000.1/, accessed on 1 August 2023). The genome was predicted to have 17,735 genes with an average gene length of 1292 bp.

At the same time, the closely related *Fusarium graminearum* genome (AACM00000000, https://www.ncbi.nlm.nih.gov/nuccore/AACM00000000.2/, accessed on 1 August 2023) was sequenced [25]. It was compared with the genomes of *Fusarium verticillioides* (AAIM02000000, https://www.ncbi.nlm.nih.gov/nuccore/AAIM00000000.2/, accessed on 1 August 2023) and *Fol* [24]. This revealed lineage-specific (LS) regions on chromosomes 3, 6, 14, and 15 of the *F. oxysporum* genome that were missing in *F. graminearum* and *F. verticillioides*. These LS regions contain genes involved in host cell wall degradation, ethylene and necrosis induction, and were shown to be upregulated during early tomato infection. As the transfer of these chromosomes could induce pathogenicity in non-pathogenic *F. oxysporum* strains, these LS regions were proposed to facilitate the transfer of pathogenicity factors among *Fusarium* species and subsequently broaden their host range [24].

More recently, the *Fol* genome was sequenced with higher coverage (66×) using Illumina HiSeq and PacBio [26]. This version of the whole-genome sequence is the current reference genome for *Fol* 4287 (QESU00000000, https://www.ncbi.nlm.nih.gov/nuccore/QESU00000000.1, accessed on 1 August 2023). This assembly is 53.9 Mb, with 499 contigs and an N_50_ scaffold length of 1.3 Mb. The largest contig is 5.7 Mb with a 47.7% GC content.

Several recent sequencing projects for other *F. oxysporum* strains with differing hosts such as flax [27], cucumber [28], cabbage [29], cowpea [30], and melon [31] have been reported. Further, Schmidt et al. described the sequencing of several strains of *F. oxysporum* f. sp. *melonis* (*Fom*), which revealed an avirulence protein that interacts with the melon resistance gene *Fom-2* [31]. The availability of *F. oxysporum* genomes for various strains has facilitated comparative genomic approaches to reveal genes involved in pathogenicity. Most recently, 35 genomes of different *Fusarium* species have been integrated into the *F. oxysporum* Pangenome Database (FoPGDB), allowing for efficient and comprehensive genomic analysis [32].

### 2.2. Transcriptomic and Secretomic Analyses

Transcriptomic and secretomic analyses of *F. oxysporum* during host infection have revealed the regulation of several pathogenicity-related genes. For instance, RNA-seq performed during host infection identified upregulated genes encoding cell wall-degrading enzymes, such as endo-polygalacturonases *PG1* and *PG5* [33]. Additionally, genes involved in synthesizing mycotoxins, such as trichothecene and fumonisin, were upregulated. Using a similar approach, Chang et al. discovered a β-lactamase-encoding gene that allowed *F. oxysporum* to infect soybean even in the presence of bacterial competitor *Burkholderia ambifaria* [34].

In a study comparing the secretomes of two strains of the banana pathogen *F. oxysporum* f. sp. *cubense* (*Foc* or *Focub*), 120 and 129 secreted proteins were identified during root infection in strains *Foc R1* and *Foc TR4*, respectively [35]. Specifically, in *Foc TR4*, a cysteine biosynthesis enzyme was found to be highly induced during root infection and was necessary for pathogenicity. In *Fol*, a secretomic analysis of acetylated proteins using LC-MS/MS revealed 32 genes that were not found in lineage-specific regions, 26 of which were upregulated during root infection [36]. These genes induced during infection provide a resource for potential reverse genetic studies to elucidate the mechanisms required for *F. oxysporum* pathogenicity.

Despite the broad range of -omic studies, an *F. oxysporum* deletion mutant for a potential gene should be generated and characterized to understand whether the gene is necessary for pathogenicity. Hence, the rest of this review will summarize the genes that have been studied in *F. oxysporum* using mutant analysis.

## 3. Molecular Dissection of *F. oxysporum* Biology

**Figure 2:**
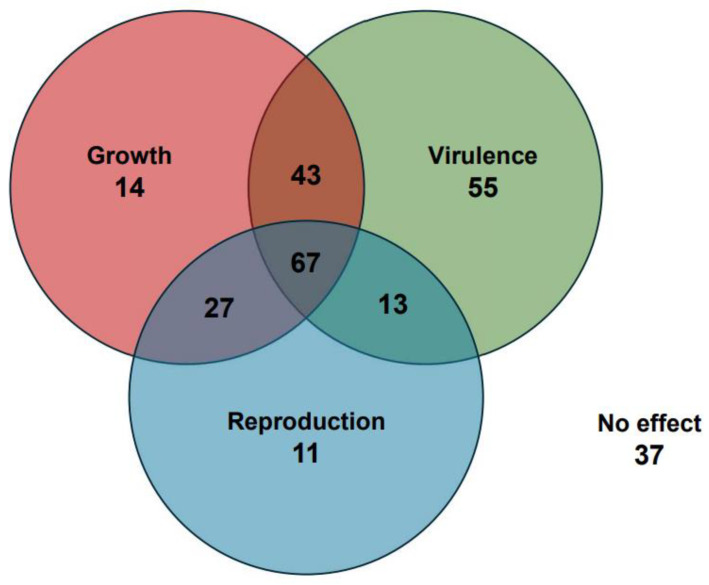
Venn diagram of *F. oxysporum* genes studied through mutant analysis. Growth includes genes affecting vegetative hyphal growth and stress tolerance. Reproduction includes genes affecting conidia and chlamydospore production. Virulence includes genes affecting pathogenicity, invasive growth, surface hydrophobicity, and fusaric acid production. A full list of genes is available in Supplementary Table S1.

**Figure 3:**
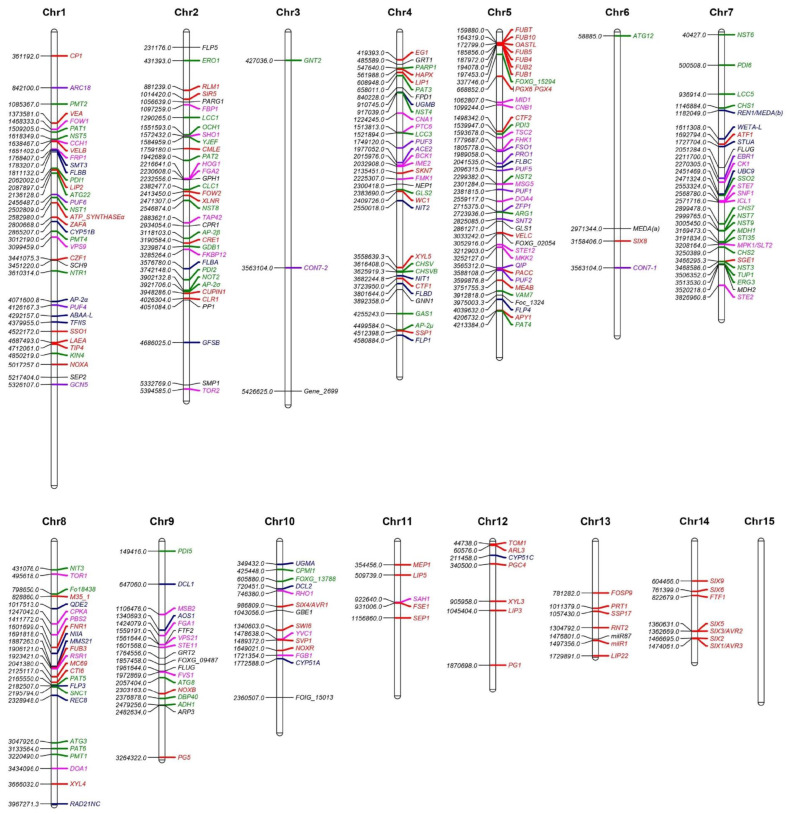
Chromosome map of *F. oxysporum* genes studied through mutant analysis. Components of central signaling pathways are labeled in pink, shared transcription factors are labeled in purple, genes involved in growth and stress tolerance are labeled in green, genes involved in reproduction are labeled in blue, and genes involved in virulence are labeled in red. The chromosomal map was drawn using MapChart. Full details are available in Supplementary Table S1.

**Figure 4:**
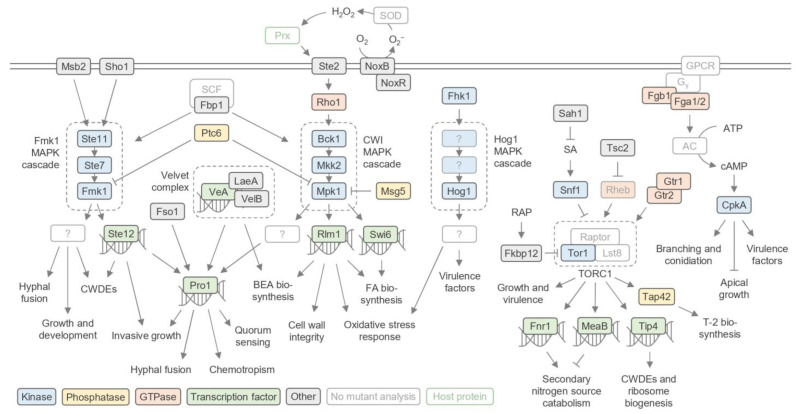
Major *F. oxysporum* signaling pathways identified through mutant analysis. CWDEs, cell wall-degrading enzymes; MAPK, mitogen-activated protein kinase; CWI, cell wall integrity; BEA, beauvericin; FA, fusaric acid; SA, salicylic acid; RAP, rapamycin; GPCR, G protein-coupled receptor; SOD, superoxide dismutase.

In this section, we describe the known functions *F. oxysporum* genes studied through mutant analysis. A comprehensive list of genes can be found in Supplementary Table S1, while an overview of the key phenotypes identified and their locations in the *F. oxysporum* genome are provided in Figure 2 and Figure 3, respectively. We begin with the signaling pathways and gene expression regulators that are shared between many aspects of *F. oxysporum* biology (Figure 4). Next, we describe the core genes involved in vegetative growth and then consider genes specific to reproductive development and virulence. Protein, gene, and mutant nomenclatures vary amongst *Fusarium* researchers. For clarity, the most consistently used system has been adopted here: proteins are denoted with the first letter capitalized (Abc1), genes are italicized with all letters capitalized (*ABC1*), and mutant alleles are denoted in lower case (*abc1*).

### 3.1. Central Signaling Pathways and Transcription Factors

#### 3.1.1. MAP Kinase Signaling

Mitogen-activated protein kinase (MAPK) signaling cascades play a central role in multiple aspects of fungal biology (Figure 4). The *F. oxysporum* MAPK Fmk1 is required for growth, virulence, and development, with essential roles regulating invasive growth, expression of CWDEs, vegetative hyphal fusion, and surface hydrophobicity (a key determinant of virulence) [37,38,39,40]. The Fmk1 cascade involves Fmk1, the MAPKK Ste7, and the MAPKKK Ste11, and is regulated by the membrane proteins Msb2 and Sho1, which act upstream of Fmk1 to promote invasive growth and cell wall integrity [41,42,43].

Another MAPK involved in *F. oxysporum* virulence is Hog1. The Hog1 pathway is activated by the histidine kinase Fhk1 and is a key mediator of virulence and oxidative stress responses [39,40,44]. The cell wall integrity (CWI) MAPK cascade—consisting of the MAPK Mpk1/Slt2, the MAPKK Mkk2, and the MAPKKK Bck1 acting downstream of the Rho-type GTPase Rho1—is required for cell wall stress resistance, hyphal fusion, and chemotrophic growth [40,43,45]. Furthermore, the MAP kinase Pbs2 is required for host perception, colonization, and virulence [39]. The serine/threonine protein kinase Ime2 controls vegetative growth, hyphal branching, conidiation, pathogenicity, and stress responses [46]. Ime2 is thought to act upstream of MAPK cascades, though its placement requires further study.

Dephosphorylation of MAPK cascade components is key to regulating their signaling activity. In *F. oxysporum*, the type 2C protein phosphatase (PP2C) Ptc6 is involved in regulating MAPKs such as Mpk1 and Fmk1 and downstream growth and virulence activities [47]. The dual-specificity phosphatase Msg5 also dephosphorylates Mpk1, as well as the MAPK Fus3 [48]. Through this dephosphorylation, Msg5 functions as a regulator of pheromone responses and cell wall integrity.

#### 3.1.2. TOR Signaling

A key regulator of growth and virulence in *F. oxysporum* is the protein complex TORC1, centered around the serine/threonine protein kinase target of rapamycin (TOR). TOR is highly conserved in eukaryotes, playing central roles in nutrient and hormone signaling networks [49,50]. Due to its importance, TOR knockout mutants are often lethal. siRNA-mediated knockdown of *TOR1* resulted in inhibited mycelial growth on potato leaves, while transgenic *TOR1* RNAi potato plants showed increased resistance to *F. oxysporum* infection, suggesting an important role for Tor1 in virulence [51]. However, chemical inhibition of TORC1 by rapamycin treatment—a process mediated by FK-506-binding protein (Fkbp12)—resulted in increased mycelial growth, suggesting a complex function of this complex [52,53]. Transcriptomic analysis has revealed that Tor1 regulates key growth and virulence-related pathways, including ribosome biogenesis and CWDEs [53].

The Gtr1–Gtr2 GTPase complex acts upstream of Tor1 to regulate its functions in growth and secondary metabolism in response to different nutrient conditions. In *F. oxysporum*, the presence of certain amino acids, most notably cysteine, causes the Gtr1–Gtr2 complex to recruit TORC1, which induces the production of T-2 toxin (T-2)—a mycotoxin harmful to humans—via the downstream phosphatase Tap42 [54].

The phytohormone salicylic acid (SA), a key modulator of the plant immune system, inhibits TOR1 by activating the adenosine monophosphate-activated protein kinase (AMPK) Snf1. Despite inhibiting TOR1, Snf1 is also required for the expression of CWDEs and is thus a key virulence factor in its own right [51,55]. To combat the inhibitory effects of SA, *F. oxysporum* produces salicylate hydroxylases (SAHs) such as Sah1 to degrade SA [51].

#### 3.1.3. GTPase Signaling

G proteins are heterotrimeric GTP-binding proteins containing α, β, and γ subunits. They transduce external signals perceived by G protein-coupled receptors (GPCRs) to a range of intracellular targets, often through activating a cyclic AMP-protein kinase A (cAMP-PKA) cascade [56]. The *F. oxysporum* G protein α subunits Fga1 and Fga2 and the β subunit Fgb1 are required for development and pathogenesis [57,58,59,60]. G protein subunit mutants displayed defects including reduced intracellular cAMP levels, reduced pathogenicity, and altered physiological features such as heat resistance, colony morphology, conidia formation, and conidia germination. Downstream of this, cAMP-dependent protein kinase A (CpkA) affects growth, morphology, root attachment, penetration, and pathogenesis [61].

In addition to heterotrimeric G proteins, the Ras-related small GTPase Rsr1 has recently been identified as an important regulator of FA biosynthesis, conidiation, and secondary metabolism [62], and the Rab family small GTPase Vps21 is required for development and virulence [63]. RAS GTPases are activated by guanine nucleotide exchange factors (GEFs). The *F. oxysporum* GEF Vsp9 acts as a GEF for Vps21 and plays an important function in endocytosis and autophagy [63]. In contrast, GTPases are inactivated by GTPase-activating proteins (GAPs). The *F. oxysporum* GAP Tsc2 acts as a negative regulator of TORC1, with loss of Tsc2 resulting in reduced growth, stress tolerance, and virulence, indicating that constitutive activation of TORC1 negatively impacts these processes [64].

#### 3.1.4. Ubiquitination

Ubiquitination is an important post-translational modification, regulating cellular processes across eukaryotes [65]. The addition of ubiquitin to target proteins is catalyzed by a series of enzymes: ubiquitin is activated by the ubiquitin-activating enzyme (E1), transferred to the ubiquitin-conjugating enzyme (E2), and finally transferred to the target protein through ubiquitin ligase (E3). Ubiquitination of substates can either regulate their activity or target them for degradation by the proteasome, forming the ubiquitin–proteasome system (UPS).

Several studies have demonstrated the importance of ubiquitination in *F. oxysporum*. The F-box protein Fbp1—a component of the SCF E3 complex—is required for pathogenesis, invasive growth, and cell wall integrity, and is believed to regulate multiple virulence-related MAPK signaling pathways [66]. Another F-box protein, Frp1, is also required for virulence, with a *frp1* mutant showing reduced expression of CWDEs and the glyoxylate cycle gene *ICL1* [67,68]. However, the direct targets of Frp1 remain unknown. Downstream of ubiquitination, Cdc48 is an essential ATPase that interacts with ubiquitinated proteins via ubiquitin-binding cofactors, including Doa1 of *Saccharomyces cerevisiae* [69]. Deletion of the *F. oxysporum* f. sp. *niveum* (*Fon*) homologue disrupts vegetative growth, conidiation, and stress tolerance [70]. Ubiquitination is a reversible modification, with ubiquitin tags removed by deubiquitinases. Deletion of Doa4, a putative deubiquitinase, also results in compromised vegetative growth, conidiation, and stress tolerance, reflecting the diverse roles ubiquitination plays in fungal biology [70].

#### 3.1.5. Other Signaling Components

Temporal and spatial changes of cytoplasmic calcium (Ca^2+^) ions play a key role in regulating the cellular and developmental responses in fungi [71]. Three Ca^2+^ channel genes, *CCH1*, *MID1*, and *YVC1*, have been identified with important roles in *F. oxysporum* biology. Cch1 and Mid1 are both required for normal vegetative growth, while Mid1 and Yvc1 contribute to sporulation [72].

In fungi, the calcium/calmodulin-dependent serine/threonine protein phosphatase complex calcineurin is involved in maintaining a diverse range of cellular processes such as growth, morphogenesis, cellular processes, stress response, and pathogenicity [73]. In *F. oxysporum*, deletion of the catalytic (Cna1) and regulatory (Cnb1) subunits demonstrated that calcineurin plays an important function in phosphatase activity and vegetative growth, virulence, and conidiation [74].

Another important regulator of *F. oxysporum* biology is Casein kinase 1 (Ck1), which negatively regulates the essential plasma membrane H^+^-ATPase Pma1 to promote alkalization of the extracellular environment and regulate hyphal growth and conidiation [75]. Ck1 also controls the hyphal chemotropism toward plant roots and pathogenicity on host plants.

*FVS1* encodes a protein with a sterile alpha motif (SAM) domain that is involved in protein–protein interactions related to signal transduction and gene regulation. Fvs1 is involved in the production of micro- and macroconidia, the development of conidiogenesis cells, conidiophores, and phialides, as well as in vegetative growth and virulence [76].

Interestingly, the mitochondrial carrier protein Fow1 is required for virulence and colonization of host tissues, but not for mycelial growth or development, in contrast to most mitochondria-localized proteins [77]. Similarly, the putative membrane protein Fpd1 contributes to virulence [78]. How Fow1 and Fpd1 fit into the known regulators of *F. oxysporum* virulence remains to be determined.

#### 3.1.6. Shared Transcription Factors and Gene Regulation Components

While some transcription factors (discussed later) regulate specific biological processes, many appear to act as ‘global regulators’, controlling multiple aspects of *F. oxysporum* biology. Con7-1 regulates a diverse range of key processes including cell wall biogenesis and remodeling, cell division, and invasive growth [79]. The C2H2 zinc finger transcription factor Czf1 plays an important function in the production of fusaric acid (FA; a virulence factor of *F. oxysporum*), secondary metabolism, conidiation, and early host infection [45]. The BAH/PHD domain-containing transcription factor Snt2 is also important for conidia production, vegetative growth, and hyphal septation of *F. oxysporum* [80], and the Zn_2_Cys_6_ domain-containing transcription factor Ebr1 contributes to growth and virulence via the regulation of virulence factors and genes involved in diverse metabolic pathways [81].

The GATA-type transcription factor Pro1 has recently been characterized at the interface of multiple signaling pathways, integrating signals from the Fmk1 and CWI MAPK cascades and the fungal-specific velvet transcription factor complex to regulate quorum sensing, hyphal fusion, and chemotropism [82]. Pro1 also acts downstream of Fso1, a regulatory protein of unknown biochemical function that is required for hyphal fusion [38]. Furthermore, the C2H2 zinc finger transcription factor Zfp1 regulates growth, conidiation, stress tolerance, and pathogenicity on *Polygonatum kingianum*, and Ace2 regulates growth, conidiation and virulence on banana, at least in part through the regulation of cell well integrity [83,84].

The Ccr4–Not complex—a multi-functional complex that regulates both transcription and translation—is similarly involved in FA biosynthesis, as well as oxidative stress tolerance, cell wall integrity, conidiation, and vegetative growth [85]. Furthermore, the histone acetyltransferase (HAT) Gcn5 plays a key role in *F. oxysporum* biology through the regulation of gene expression. Gcn5 is a member of the GNATs family of type A HATs and regulates the apical deposition of the cell wall material, as well as tolerance to heat, salt, and cell wall inhibitors [86].

In fungi, the velvet complex is a key regulator of development and the biosynthesis of secondary metabolites, acting through the modulation of chromatin accessibility and gene expression [87,88]. The velvet family proteins VeA and VelB interact with the non-velvet protein LaeA in the absence of light to form the heterotrimeric velvet complex [87,89]. Mutation analysis indicates that VeA and LaeA have partially overlapping functions in the development of hyphae and the conidiation and light response of *F. oxysporum* [90,91,92].

Post-transcriptional regulation also plays a key role in the control of gene expression. The Pumilio protein family (PUF) of RNA-binding proteins is important for the regulation of mRNA stability and translation in eukaryotes [93]. PUF proteins have diverse roles in *F. oxysporum* biology, with Puf1-4 regulating vegetative growth, Puf1-6 involved in macroconidia development, and Puf1 required for full virulence [94]. In particular, Puf1 interacts with the actin-related protein 2/3 (ARP2/3) complex via the complex component Arc18. Arc18 itself plays an important role in *F. oxysporum* virulence and ATP generation in mitochondria.

Small RNAs also play important roles in the regulation of *F. oxysporum* virulence at the translational level. Fungi produce microRNA-like RNAs (milRNAs) that are similar to plant and animal microRNAs in structure, playing important functions in different biological processes [95]. Deletions of the Argonaute protein Qde2, the Dicer-like proteins Dcl1 and Dcl2, and the exonuclease Qip—all components of milRNA processing pathways—variously impacted growth, conidiation, and virulence [96,97]. In particular, Qde2 upregulates the expression of the milRNA gene *milR87*, which contributes to virulence by suppressing the avirulence gene *FOIG_15013* [97]. In addition, the *milR106* is important in promoting conidiation, oxidative stress tolerance, and virulence [98].

### 3.2. Genes Involved in Vegetative Hyphal Growth and Stress Tolerance

This sub-section deals with the genes involved in the vegetative hyphal growth and stress tolerance of *F. oxysporum*, although many of these genes also impact virulence and reproduction.

#### 3.2.1. Protein Post-Translational Modifications (PTMs)

Post-translational modifications (PTMs) are vital for the activity and regulation of proteins in all aspects of cellular biology. Protein O-mannosylation is a PTM conserved in eukaryotes that is catalyzed by protein O-mannosyltransferases (PMTs). In *F. oxysporum* f. sp. *cucumerinum*, mutants of *PMT* genes such as *PMT1, PMT2*, and *PMT4* show retarded growth, reduced conidiation, cell wall defects, attenuated virulence, and altered ER stress response [99]. Pmt1 targets nuclear proteins and components of the protein folding machinery. Pmt2 also regulates protein folding as well as cell wall synthesis. Pmt4 acts on proteins in secretory pathways, notably the GPI anchoring pathway involved in polarized growth.

Protein palmitoylation, another PTM, is catalyzed by a group of palmitoyl transferases (PATs). In the *Fon* genome, six PAT genes play key roles in conidiation, conidial morphology, stress response, and vegetative growth [100]. Among them, *PAT1*, *PAT2*, and *PAT4* regulate virulence. In an in vivo assay, Pat2 palmitoylated subunits of the AP-2 complex, a heterotetrameric endocytic cargo-binding adaptor. This palmitoylation contributes to the interaction and stability of the core subunits and is required for vegetative growth, cell wall integrity, asexual reproduction, and virulence.

Glycosylation is another PTM with important roles. Nucleotide sugar transporters (NSTs) link the synthesis of nucleotide sugars and glycosylation in the ER or Golgi and function as antiporters of nucleotide monophosphates [101,102]. The *Fon* genome contains nine *NST* genes that show distinct functions in vegetative growth, cell wall stress response, asexual production, and virulence [103]. In particular, Nst2 and Nst3 are essential for virulence, with Nst2 mainly affecting host colonization. Nst2 acts as a UDP-galactose transporter and interacts with the protein disulfide isomerase Pdi1 and the oxidoreductase Ero1, important regulators of disulfide bond formation.

Poly(ADP-ribosyl)ation (PARylation) is another important PTM in eukaryotes and is catalyzed by poly(ADP-ribose) polymerases (PARPs) and hydrolyzed by poly(ADP-ribose) glycohydrolases (PARGs). *F. oxysporum* Parp1 is required for pathogenicity—while its targets remain unknown, Parp1 is phosphorylated in vitro by the kinase Kin4 to enhance PARP activity [104].

#### 3.2.2. Vesicle Trafficking

Soluble N-ethylmaleimide-sensitive factor attachment protein receptors (SNAREs) are conserved in fungi, animals, and plants and have a vital function in vesicle trafficking. The *F. oxysporum* Vam7 protein contains SNARE and Phox homology (PX) domains [105]. Vesicle trafficking mediated by Vam7 is critical in vegetative growth, asexual reproduction, and host infection of *F. oxysporum.* Vam7 also regulates the sensitivity of the fungus to salt and osmotic stress and cell wall stresses. In addition, the SNARE proteins Sso1 and Sso2 contribute to growth, conidiation, and virulence by forming complexes with the SNARE proteins Sec9 and Snc1 [106]. Sso1 appears to regulate exocytosis at the growing hyphal apex, while Sso2 is mainly expressed in older hyphae.

#### 3.2.3. Autophagy

Autophagy appears to play key roles in multiple aspects of *F. oxysporum* biology, with mutations in components of the autophagy pathway (*ATG* genes) resulting in severe defects. The ubiquitin-like protein Atg8—an autophagy component required for the formation of the autophagosome—mediates nuclear degradation after hyphal fusion and plays a general function in the control of nuclear functions [107]. Atg3 regulates the conidiation, hyphal growth, and virulence of *F. oxysporum* [108]. Atg22 is important in the formation of autophagosomes and regulates the hyphal development, conidiation, and pathogenicity of *F. oxysporum* [109]. Atg12 affects the expression of genes involved in pathogenicity, vegetative growth, and morphological features under various stresses [110].

#### 3.2.4. Metabolism and Nutrient Acquisition

As expected, mutations in genes related to primary metabolic pathways can result in compromised growth, virulence, and development. Loss of the isocitrate lyase *ICL1*—an enzyme in the glyoxylate cycle—resulted in reduced growth on various carbon sources [68]. Expression of *ICL1* and other genes involved in glyoxylate metabolism is regulated by the CCCH-type zinc finger-containing protein Dbp40, the deletion of which also results in decreased growth and virulence [111]. Tup1, a component of the Tup1–Cyc8 transcriptional corepressor complex, is also involved in mycelial growth, conidia development, and virulence through the regulation of numerous metabolic pathways, including the tricarboxylic acid (TCA) cycle [112]. Similarly, deletion of genes encoding the TCA cycle enzyme malate dehydrogenase (*MDH1/2*) compromised mycelial growth, conidiation, and virulence [112].

Other genes related to diverse metabolic pathways have also been studied in *F. oxysporum*. Disruption of the putative argininosuccinate lyase Arg1, a key enzyme in arginine biosynthesis, resulted in reduced vegetative growth and virulence [113]. The alcohol dehydrogenase gene *adh1* is highly expressed during hypoxia, and a small deletion in the *ADH1* gene resulted in reduced growth in hypoxic conditions and delayed virulence on tomato [114]. Deletion of the predicted glycogen debranching enzyme-encoding gene *GDB1* resulted in compromised virulence and vegetative hyphal growth—although deletion of other genes involve in glycogen biosynthesis and catabolism caused no notable phenotype [115]. Thiamine (vitamin B1) is an essential vitamin that functions as a major cofactor of enzymes that are critical for carbohydrate metabolism, including the pentose–phosphate cycle, the citric acid cycle, and glycolysis [116]. The *F. oxysporum* stress-induced gene *STI35* functions in thiamine biosynthesis [117]. In yeast, orthologs of *STI35* are highly expressed and depend on transcriptional repression by thiamine [118]. Mutation analysis of *F. oxysporum STI35* has revealed that Sti35 plays an important role in thiamine biosynthesis and in oxidative stress tolerance [119]. Finally, deletion of YjeF, a homolog of an *Escherichia coli* cellular metabolism damage-repair enzyme, significantly reduced growth, sporulation, and virulence [120].

Nitrate assimilation is also central to fungal growth and development. *F. oxysporum* strains such as *Fol* can utilize nitrate as the only source of nitrogen. Mutation analysis has shown that the predicted nitrate reductase gene *NIT1* and high-affinity nitrate/nitrite transporter gene *NTR1* are critical for nitrate assimilation of *Fol* under aerobic and anaerobic conditions, but this is not essential for virulence [121].

#### 3.2.5. Cell Wall Biogenesis, Integrity, and Remodeling

Fungal cell walls are essential for their survival. Enzymes involved in the synthesis and maintenance of the cell wall are crucial for *F. oxysporum* growth and development. Mutant analysis has also indicated that cell wall integrity plays a key role in *F. oxysporum* virulence: deletions of genes involved in cell wall architecture have resulted in reduced virulence, including those encoding chitin synthases (*CHS2*, *CHS7*, *CHSV*, and *CHSVB*), an ergosterol biosynthesis gene (*ERG3*), a β-1,3-glucanosyltransferase (*gas1*), an *N*-acetylglucosaminyl transferase (*GNT2*), protein O-mannosyltransferases (*PMT1*, *PMT2*, and *PMT4*), a phosphomannose isomerase (*CPMI1*), a β-1,3-glucan synthase subunit (*GLS2*), and a putative α-1,6-mannosyltransferase (*OCH1*) [99,122,123,124,125,126,127,128,129,130]. The putative UDP-galactopyranose mutase (UGM)-encoding genes *UGMA* and *UGMB* are involved in the production of galactofuranose-containing sugar chains, affecting vegetative growth, pathogenesis and conidiation of *F. oxysporum* [131]. These studies have revealed a number of mechanisms through which cell wall integrity may contribute to virulence, including tolerance to host-associated stresses and defense compounds, recognition of external signals, and invasive growth and reproduction.

#### 3.2.6. Stress Tolerance and Defense

During pathogen colonization, plants produce reactive oxygen species (ROS) as both immune signals and direct anti-microbials. To protect themselves from ROS damage, fungal pathogens produce a variety of antioxidant enzymes to detoxify the host environment, including catalases and peroxidases. In *F. oxysporum*, deletion of predicted catalase- (FOXG_15294), peroxidase- (FOXG_13788) and catalase-peroxidase- (FOXG_17180) encoding genes resulted in reduced virulence on tomato [132]. Expression of antioxidant genes in *Fol* is controlled by the serine/arginine protein kinase Srpk1, which is deacetylated upon ROS exposure, allowing translocation of Srpk1 to the nucleus.

Laccases—copper-containing phenol oxidases that catalyze the oxidation of phenolic compounds and reduce molecular oxygen to water—are also important for the protection of fungal pathogens against toxic plant compounds [133]. In *F. oxysporum*, loss of the laccase genes *LCC1* and *LCC3*, though not *LCC5*, results in more sensitivity to toxic compounds and oxidative stress [134]. Laccase activity depends upon the predicted chloride channel Clc1, with deletion of *CLC1* resulting in reduced laccase activity and delayed virulence on tomato [135].

Fungal plant pathogens also face toxic compounds produced by microbial antagonists, including β-lactam antibiotics. During soybean infection, *F. oxysporum* employs the β-lactamase-encoding gene *Fo18438* to protect against β-lactam antibiotics secreted by microbial competitors [34]. *Fo18438* is upregulated in the presence of *Burkholderia ambifaria*—a plant growth-promoting bacterium that inhibits *F. oxysporum* colonization [136].

### 3.3. Genes Involved in Reproduction

Under favorable conditions, *F. oxysporum* can produce microconidia, macroconidia, or chlamydospores [137]. These spores facilitate efficient fungal propagation and reproduction. Upon host infection, single or double-celled microconidia formed inside host tissues from phialides can germinate and grow in adjacent cells, while multicellular macroconidia predominantly form on the surface of infected tissue from conidiophores and can become airborne to spread to other hosts [138]. Finally, single or double-celled chlamydospores can be formed at the tips of macroconidia under nutrient-limiting conditions. They can overwinter in soil for long periods [138]. This subsection highlights genes that are primarily necessary for *F. oxysporum* reproduction.

#### 3.3.1. Cell Division

Knocking out genes involved in cell division is anticipated to negatively affect conidiation. Indeed, deleting components of the mitotic cohesin complex such as *RAD21* and *REC8* diminished conidial germination under cell cycle stress conditions [139]. However, conidiation was unaffected in these mutants, suggesting that other specific regulators are present to regulate conidiation.

#### 3.3.2. Transcriptional Regulators of Conidiation

Several genes encoding transcriptional regulators of reproduction in other fungi also regulate conidiation in *F. oxysporum*. From an insertional mutagenesis screen, Ren1 was identified as the *rensa* mutant of *F. oxysporum*, which exhibited reduced micro- and macroconidia formation [140]. Ren1 shows close homology to transcriptional regulators of conidiation in *Magnaporthe grisea* (Acr1) and *Aspergillus nidulans* (MedA). Ren1 and Aba1, a homolog of *A. nidulans* transcription factor AbaA, were suggested to be regulated by the transcription elongation factor TFIIS in *F. oxysporum* [141]. TFIIS deletion strains exhibited reduced *REN1* and *ABA1* expression and overall reduced conidiation and virulence.

In *A. nidulans*, AbaA is a component of the central regulatory pathway (CRP) required for conidiophore development and subsequent spore germination [142]. The CRP also consists of the transcription factors BrlA and WetA. In *F. oxysporum*, the homologs Aba1 and WetA-L are required to produce conidia, phialides, and chlamydospores [92]. BrlA has no known homolog in *F. oxysporum*, but MedA(a), an ortholog of Ren1, has a similar function to BrlA, being required for conidiophore and phialide development and regulating *ABA1* expression. Similarly, deleting the developmental transcriptional regulator StuA in *F. oxysporum* reduced the expression of *MEDA(a)* and *ABA1*, leading to reduced micro- and macroconidia. In *A. nidulans*, the upstream developmental activators (UDAs) flbB, flbC, and flbD control conidiation by regulating the expression of *BrlA* [143]. However, the expression of CRP components in *F. oxysporum*, such as *MEDA(a), ABA1*, and *WETA-L*, were not affected by UDA deletion, suggesting a species-specific function of the UDAs [92]. Nevertheless, deleting *F. oxysporum* FlbB, FlbC, and FlbD resulted in dysregulated conidiation.

#### 3.3.3. Post-Transcriptional Regulators

Along with transcriptional regulation, *F. oxysporum* mRNA stability and protein modification can control reproduction. In *Neurospora crassa*, the argonaut protein Qde2 and dicer Dcl1 were found to regulate virulence and microconidiation. Deleting these RNAi components simultaneously in *F. oxysporum* led to decreased levels of conidiation-regulating genes such as *STUA* and *NIIA*, which suggests a role of mRNA degradation in promoting conidiation [97]. Additionally, the deletion of several RNA-binding Pumilio proteins (*PUF2-6*) required for RNA processing resulted in reduced vegetative growth and decreased macroconidiation [94]. Several genes of the SUMOylation pathway involved in protein modification are required for vegetative growth and tolerance to chemical stressors; deleting *UBC9, MMS21, SMT3*, and *AOS1* produced smaller macroconidia and decreased microconidiation [144]. Similarly, deleting palmitoylation pathway components *PAT3*, *PAT5*, and *PAT6* reduced mycelial growth and dysregulated macro- and microconidiation [100]. This was suggested to occur due to the dysregulation of palmitoylating AP-2 complex components, which are known regulators of fungal virulence, cell wall integrity, and conidiation.

#### 3.3.4. Nutrient Metabolism

The availability of nutrients in the environment is critical in determining whether *Fusarium* species can reproduce [145]. Hence, genes involved in nutrient metabolism are necessary for regulating conidiation. Transcriptomic analyses in the previously mentioned *ren1* and *stuA* revealed that the nitrite reductase gene *NIIA* was downregulated in *F. oxysporum* [146]. Deleting *NIIA* resulted in reduced macroconidia production like *ren1* and *stuA* mutants. Interestingly, the nitrite-reducing activity of *NIIA* was not required for conidiation but was speculated to be necessary for producing nitric oxide, a byproduct that promotes conidiation. Expectedly, the nitrate reductase gene *NIT1* deletion mutant also exhibited decreased macroconidia production, exemplifying the importance of nitrogen metabolism toward conidiation [121].

Aside from nitrogen, the nucleotide sugar transporters (NSTs) involved in nucleotide sugar synthesis and glycosylation are required for vegetative growth, virulence, and conidiation [103]. Similarly, the sterol 14*α*-demethylases (*CYP51A* and *CYP51B*) necessary for ergosterol production seemed to negatively regulate conidiation in *F. oxysporum* [147]. Likewise, deleting the human lysine deacetylase *SIRT5* ortholog (*SIR5*) resulted in enhanced virulence and conidiation in the host [148]. In *F. oxysporum, SIR5* was suggested to reduce conidial germination through the restriction of ATP synthesis by inhibiting the pyruvate dehydrogenase complex and repressing the expression of citric acid components.

#### 3.3.5. Cell Wall Production and Stability

The proper composition of fungal cell walls consisting of glucans, glycoproteins, and chitin is integral to fungal growth, virulence, and reproduction [149]. In microconidia, the content of mannose, a cell wall component involved in stability, is decreased compared to mycelium [150]. This likely suggests that genes regulating the production and stability of cell walls are important for *F. oxysporum* reproduction. Indeed, deleting the UDP-galactopyranose mutase (*UGMB*) and galf-transferase (*GFSB*), required for cell wall stability via facilitating galactofuranose biosynthesis, led to a reduction in virulence and conidiation [131]. The *ugmA gfsB* double mutant displayed stronger conidiation deficiencies and decreased vegetative growth. Further, RNAi of the fasciclin-like protein (FLP) family involved in cell wall stability in plants and virulence of *Magnaporthe oryzae* had greatly diminished conidiation and host colonization in *F. oxysporum* [151].

#### 3.3.6. Virulence Genes Affecting Conidiation

In some instances, knocking out genes studied in the primary context of virulence also affected conidiation. For example, deleting the yeast MAPK pheromone response pathway components *STE12* and *FMK1* in *F. oxysporum* led to increased conidiation [152,153]. As *ste12 fmk1* also exhibited decreased virulence, the roles of Ste12 and Fmk1 likely vary depending on nutrient availability in the infected plant host. Further, when the velvet protein complex transcription factors involved in mycotoxin production, *VEA, VELB, VELC*, and *LAEA*, were deleted, microconidiation was also dysregulated [90]. Although all velvet deletion mutants had attenuated virulence, the number and morphology of microconidia differed among mutants: *velB* and *velC* had increased microconidia, while this was decreased in *laeA. veA* also had increased microconidia but had a strikingly elongated morphology. Seemingly, the velvet proteins regulate the conidiation of *F. oxysporum* in a complex manner. Finally, deleting the small, secreted protein (*SSP1*) enhanced virulence and conidiation [154]. At the same time, Ssp1 is secreted and is likely recognized by the host as a pathogen-associated molecular pattern (PAMP), negatively affecting *F. oxysporum* virulence.

### 3.4. Genes Involved in Virulence

*F. oxysporum* employs diverse signaling modules to facilitate invasion and colonization of host tissues. This sub-section deals with components involved in the pathogenesis mechanisms, detailing the regulated processes we understand, as well as the virulence-specific transcription factors controlling them.

#### 3.4.1. Chemotrophic Growth

Chemotropism plays a key role in the initiation of fungal pathogenesis, allowing hyphae to elongate in the direction of potential host tissue. In *F. oxysporum*, chemotrophic growth depends on the NADPH oxidase B (NoxB) complex, with the complex subunits NoxB and NoxR both required for chemotrophic growth and full virulence [155]. The NoxB complex, in coordination with secreted superoxide dismutase (SOD), mediates the synthesis of ROS that activate peroxidases (Prx) secreted by the plant roots. Prx activation leads to the activation of the cell wall integrity (CWI) MAPK cascade via the G protein-coupled receptor Ste2, which coordinates growth along the Prx activity gradient [43].

#### 3.4.2. Fusaric Acid (FA) and Nitric Oxide (NO) Production

An important *F. oxysporum* pathogenicity factor is the production of fusaric acid (FA or FSA), a phytotoxin produced by numerous *Fusarium* species. While the function of FA is not fully understood, evidence suggests that it plays a role in cell membrane damage and in chelating metal ions [156,157]. FA biosynthesis in *F. oxysporum* is controlled by the FA biosynthetic (*FUB*) cluster of twelve genes [158], which appears to be well conserved among *F. oxysporum* strains [159]. Targeted deletions of six genes (*FUB1-5* and *FUB10*) resulted in reduced FA biosynthesis, invasive growth, and disease severity, indicating their importance in *F. oxysporum* pathogenicity [157,159]. Another key component of FA production is the major facilitator superfamily (MFS) transporter protein FubT. *FUBT* is required for FA secretion in *F. oxysporum* f. sp. *vasinfectum* (*Fov*), but its disruption also resulted in reductions in FA biosynthesis and fungal resistance to exogenous FA [160], alluding to complex regulation of FA homeostasis.

NO has been widely implicated in plant–pathogen interactions, with important functions as a signaling molecule in growth, development, and stress responses of both plants and fungi [161]. Recently, NO was shown to play a key role in the pathogenicity of *F. oxysporum* f. sp. *cubense* (*Foc* or *Focub*) on banana [162]. Meta-transcriptomic analysis identified the upregulation of NO biosynthesis and detoxification genes upon infection, and deletion of two NO biosynthesis genes encoding putative NAD(+)-dependent formate dehydrogenase and nitrite reductase resulted in compromised virulence.

#### 3.4.3. Cell Wall Degrading Enzymes (CWDEs)

As with many fungal plant pathogens, *F. oxysporum* secretes a large repertoire of cell wall-degrading enzymes (CWDEs), including polygalacturonases (PGs), xylanases, glycosidases, and proteases. Initial studies have shown these enzymes to be secreted during infection, but their contributions to virulence have been difficult to determine. The PG genes *PG1*, *PGX4*, *PG5*, and *PGX6*, the xylanase genes *XYL3*, *XYL4*, and *XYL5*, the protease gene *PRT1*, and the lipase genes *LIP1*, *LIP2*, *LIP3*, *LIP5*, and *LIP22* have all been deleted in *Fol* without any impact on virulence [163,164,165,166,167,168,169]. The lack of virulence phenotypes may be in part due to the functional redundancy of many CWDE genes—single deletions of *PG1* and *PGX6* had no impact of virulence, but a *pg1pgx6* double mutant showed reduced pathogenicity on tomato [163]. Furthermore, knockouts of genes regulating multiple CWDEs generally have a clearer impact on virulence, such as the carbon catabolite repressor *SNF1* and the lipase and cutinase transcriptional regulators *CTF1* and *CTF2* [55,168]. Genetic redundancy poses a major challenge for studies utilizing mutant analysis, particularly in forward genetic screens that largely characterize single mutants. Future studies will require alternative strategies to overcome redundancy, including generating higher-order mutants, employing RNAi or CRISPR for efficient knockdown/knockout of multiple genes, and utilizing overexpression of target genes.

In some cases, however, a single deletion was sufficient to reduce virulence, such as for the PG-encoding gene *PGC4* in *Focub* and the glycosidase-encoding gene *EG1* in *Fol* [170,171], suggesting their key roles in host cell wall degradation. 3-carboxy-cis,cis-muconate lactonizing enzyme (Cmle) is also required for full virulence and is believed to play an important role in the breakdown of lignin and phenolic compounds secreted by the host during infection to strengthen its cell wall [172]. Interestingly, deletion of the glycosidase gene *FOIG_15013* increased virulence, potentially because the enzyme releases fragments from the host cell wall that are recognized by the host as damage-associated molecular patterns (DAMPs), inducing an immune response [97]. To combat this, *FOIG_15013* is suppressed during infection by the microRNA-like RNA *milR87*.

#### 3.4.4. Xylem Effectors: Secreted in Xylem (SIX) Proteins

Numerous secreted effector proteins have been identified from the xylem sap of tomato plants infected with *Fol* and are thus named secreted in xylem (SIX) proteins [173,174,175]. Homologous proteins have since been identified in other strains, though the number of *SIX* genes varies between and even within *formae speciales* [176]. Mutant analysis has shown that many SIX proteins function as key virulence factors—deletions of *SIX1*, *SIX3*, *SIX4*, *SIX5*, *SIX6* and *SIX8* resulted in reduced pathogenicity of various *formae speciales*, although *SIX2* and *SIX9* knockouts produced no virulence defects [162,177,178,179,180,181,182,183,184,185,186,187,188]. While the specific functions of SIX proteins are largely unknown, some studies point to roles in inhibiting host defense pathways, such as jasmonic acid signaling and the hypersensitive response (HR) [182,184]. In *F. oxysporum* f. sp. *conglutinans* (*Focon*), *SIX8* is physically linked with the effector gene *PSE1* [188]. The *SIX8–PSE1* linkage pair is required for virulence on *Arabidopsis* and is thought to act by suppressing the phytoalexin camalexin.

Despite a clear contribution to virulence, certain *SIX* genes are known to participate in gene-for-gene interactions with resistance (*R*) genes in the respective host plants, causing recognition by specific host cultivars. In *Fol*, *SIX4* triggers resistance mediated by the tomato *R* gene *IMMUNITY* (*I*), *SIX3* and *SIX5* trigger *I-2*-mediated resistance, and *SIX1* triggers *I-3* resistance [12,173,175,183]. These *SIX* genes are thus also referred to as avirulence (*AVR*) genes (*SIX4* is *AVR1*, *SIX3* is *AVR2*, and *SIX1* is *AVR3*). Similarly, *SIX6* deletion in *Fon* increased virulence on watermelon, although the host resistance gene involved is yet to be identified [185]. These avirulent properties have also played an important role in race discrimination. *Fol* race 2 is thought to have evolved from race 1 through a deletion of the *SIX4/AVR1* gene, allowing it to evade *I*-mediated resistance. Race 3 then emerged through a mutation in *SIX3/AVR2*, circumventing *I-2*-mediated resistance.

#### 3.4.5. Effectors in Pathogenicity and Suppression of Host Defenses

In addition to CWDEs and SIX proteins, mutant analysis has identified several other secreted proteins with key roles in *F. oxysporum* virulence, many of which operate by inhibiting host immunity. In *Fol*, the metalloprotease Mep1 and serine protease Sep1 contribute to virulence by cleaving host chitinases, which function in host resistance by attacking the fungal cell wall [189]. Similarly, the *Focub* metalloprotease M35_1 suppresses chitinase activity, as well as inhibiting the hypersensitive response (HR) [190]. The secreted enzyme O-acetylhomoserine (thiol)-lyase (Oastl) is thought to interfere with host biosynthesis of cysteine, a precursor to numerous defense compounds [35], while the Cupin domain-containing protein Cupin1, the α-pheromone-like protein Pp1, and the small secreted protein Ssp17 also inhibit host immune responses [191,192,193]. *Fol* also secretes the tomatinase enzyme Tom1, which degrades the tomato antimicrobial defense compound α-tomatine [194]. The *Focub* effector Fse1 regulates virulence via a direct interaction with a host MYB transcription factor involved in the induction of cell death [195]. Furthermore, the *Fol* secreted protein SVP1 suppresses host defenses by relocating the tomato defense protein SlPR1 from the apoplast to the nucleus, preventing its defense signaling activity [196]. Interestingly, SVP1 is protected from ubiquitin-mediated degradation in both the fungal and plant cells by acetylation catalyzed by the sine acetyltransferase ARD1.

Like other pathogenic fungi, *F. oxysporum* employs cross-kingdom RNA interference during pathogenesis. The microRNA-like RNA *milR1* acts as a secreted effector in *Fol*, contributing to virulence by suppressing a tomato resistance gene [197]. Extracellular ATP also plays a role in suppressing host immune responses—mutants lacking the ATP synthase gene *ATP SYNTHASEα* were unable to suppress expression of host sugar transporters, which are believed to contribute to host immunity by starving invading pathogens [198].

A number of other putative secreted effectors contribute to virulence through unknown mechanisms, including the aminopeptidase Apy1 [187], the ribonuclease Rnt2 [199], the secreted proteins Fosp9 and Foc 1324 [200,201], and the small secreted proteins Mc69 and Cep28 [202,203]. Cp1, a secreted cerato-platanin (CP) protein, is required for penetration of *Focub* [204]. The secretion of these effectors requires the creation of secretory vesicles in a process orchestrated by the Arf family proteins, including Arf, Arl, and Sar proteins. In *F. oxysporum*, *ARL3* deletion resulted in reduced virulence, while viable cells could not be obtained from *ARF1* deletion, indicating the essential roles of these proteins [205].

#### 3.4.6. Virulence-Specific Transcription Factors and Small RNA Regulation

*F. oxysporum* employs many transcription factors to regulate the expression of virulence factors. While several transcription factors regulating growth and development are also required for full virulence, this section will focus on those specific to pathogenesis.

CWDEs are regulated by a range of transcription factors. Ctf1 and Ctf2 exert both positive and negative regulation of lipase and cutinase genes [168], while XlnR is a transcriptional activator of xylanase genes, but is not required for virulence [206]. Tip4 acts downstream of Tor1 to promote expression of CWDEs, as well as genes related to ribosome biogenesis [53]. Clr1 activates expression of cellulolytic enzymes, but its deletion also led to increased expression of other virulence factors, including other CWDEs, resulting in increased virulence [207]. PacC and Cre1 act as negative regulators of virulence, with PacC suppressing the expression of polygalacturonase and pH-responsive virulence genes, and Cre1 suppressing CWDE and nutrient acquisition genes [208,209].

FA production is controlled in part by the C2H2 zinc finger transcription factor Czf1, which activates transcription of *FUB* genes [210]. Downstream of the CWI MAPK cascade, Rlm1 and Swi6 also regulate FA biosynthesis, and Rlm1 further functions in cell wall integrity, beauvericin biosynthesis, and oxidative stress responses [211,212]. The core genome transcription factor Sge1 regulates *SIX* gene expression and is required for pathogenicity and conidiation, but not for colonization or penetration [213]. *SGE1* itself is regulated by Ftf1 and 2, which positively regulate virulence during early infection [214,215]. The homeodomain transcription factor Ste12 acts downstream of the Fmk1 cascade to regulate invasive growth and expression of CWDEs [152], while unknown transcription factors must affect the other functions of the Fmk1 cascade. In addition, the Zn(II)2Cys6 transcription factor Fow2 and the PHD finger-containing transcription factor Cti6 are also important regulators of *F. oxysporum* virulence, although their targets remain unknown [216,217].

Many transcription factors have characteristic roles in responding to environmental conditions during infection. The transcription factor Wc1 mediates photoreception in *F. oxysporum*, regulating surface hydrophobicity, carotenoid biosynthesis, and hyphal growth in response to UV light [218]. While *wc1* is not required for pathogenesis on tomato, it is a key virulence determinant during infection of immunocompromised mice. During infection, phytopathogenic fungi experience nutrient limitation, including nitrogen and metal irons. The GATA transcription factor Fnr1/AreA and the bZIP transcription factor MeaB act downstream of Tor1 to coordinate the regulation of nitrogen catabolism during infection [52,219]. Fnr1 promotes the catabolism of secondary nitrogen sources under the nitrogen-poor conditions present during early infection, while MeaB suppresses this response when preferred nitrogen sources are present. The bZIP transcription factor HapX contributes to virulence by regulating iron homeostasis [220], while ZafA is induced in response to zinc-poor conditions to induce expression of zinc transporters [221]. The transcription factors Atf1 and Skn7 are involved in the oxidative stress response, likely by inducing expression of antioxidant genes such as catalases and peroxidases to protect against ROS damage in the host tissues [222,223].

## 4. Conclusions and Future Directions

Fusarium wilt is a widespread and devastating disease that causes huge damage to crop plants globally. The last two decades have greatly expanded our molecular understanding of *Fusarium oxysporum* biology and its interactions with host species. Approximately 200 genes have now been studied through mutant analysis, identifying key components of diverse biological processes and elucidating the signaling framework. However, with over 17,000 predicted genes in the *F. oxysporum* genome [24], much work remains to further our understanding of this important fungal pathogen. Most signaling pathways remain incomplete, and many key components in fundamental cellular processes have yet to be studied. In particular, the regulation of *F. oxysporum* development and reproduction remains poorly understood. Furthermore, while the virulence mechanisms of *F. oxysporum* have been better studied, many of the genetic components are still missing.

Enhancing our molecular knowledge of these processes will be vital for the development of novel disease control strategies. In particular, mutant analysis provides a useful approach to identifying effective targets of RNAi silencing-based technologies such as host- and spray-induced gene silencing (HIGS/SIGS) to inhibit pathogen growth or virulence. Furthermore, a deeper understanding of *F. oxysporum* pathogenesis will assist the development of targeted crop engineering strategies to enhance disease resistance. However, such strategies will have to consider the considerable genetic diversity within and between *F. oxysporum formae speciales* and the diverse virulence strategies employed by different strains.

Recent advances in genetic engineering technologies such as CRISPR are now being applied to accelerate gene discovery and characterization. However, challenges remain. The intricate crosstalk among different signaling pathways and genetic redundancy make it difficult to dissect gene function through single mutant analysis. Mutant lethality is also a major difficulty in studying genes with vital roles in *F. oxysporum* biology, many of which may be of particular interest for disease control. Future work will need to overcome these challenges by employing alternative approaches, including multiple gene knockout, RNAi-based gene knockdown, and overexpression analysis. Furthermore, detailed studies must combine genetic analysis with cell biology and biochemical tools to develop a more complete understanding of *F. oxysporum*.

## Data Availability

No new data were created or analyzed in this study.

## Supplementary Materials

The following supporting information can be downloaded at: https://www.mdpi.com/article/10.3390/pathogens13100823/s1, Supplementary Table S1: List of *Fusarium oxysporum* genes studied by mutant analysis. References [224,225,226,227,228,229,230] are cited in the Supplementary Materials.
